# What Matters Most: ACP Evolving in Practice

**DOI:** 10.1093/geroni/igab046.1908

**Published:** 2021-12-17

**Authors:** Kevin Valadares

**Affiliations:** University of Southern Indiana, Evansville, Indiana, United States

## Abstract

The University of Southern Indiana (USI) GWEP uniquely embeds Area Agencies on Aging (AAA) care coordinators within primary care settings to invite the participation of aging patients in advance care planning (ACP), among other health interventions. Two subsequently developed features of the USI GWEP’s ACP initiative emerged to address the What Matters metric of the 4Ms: 1) Patients are invited to engage in What Matters Most conversations through multiple touchpoints that frame Medicare Wellness Visits with a Deaconess provider and introduce a free, online ACP platform, Prepare for Your Care. 2) Provider, patients and families are supported in having ACP conversations with the dedication of a new Advance Care Planning facilitator position. Certified in Respecting Choices and jointly funded by the GWEP and Deaconess, the ACP facilitator supports individuals in navigating these essential healthcare conversations about balancing quality care with quality of life.

